# Polyphenol-rich tart cherries (*Prunus Cerasus, cv* Montmorency) improve sustained attention, feelings of alertness and mental fatigue and influence the plasma metabolome in middle-aged adults: a randomised, placebo-controlled trial

**DOI:** 10.1017/S0007114522000460

**Published:** 2022-12-28

**Authors:** Rachel Kimble, Karen M. Keane, John K. Lodge, William Cheung, Crystal F. Haskell-Ramsay, Glyn Howatson

**Affiliations:** 1 Faculty of Health and Life Sciences, Northumbria University, Newcastle-upon-Tyne, UK; 2 Population Health Sciences Institute, Newcastle University, Newcastle upon Tyne, UK; 3 School of Science and Computing, Galway-Mayo Institute of Technology, Galway, Ireland; 4 Water Research Group, School of Environmental Sciences and Development, Northwest University, Potchefstroom, South Africa

**Keywords:** Anthocyanins, Cerebral blood flow, Sleep, Sustained attention, Mental fatigue, Bond–Lader, Metabolomics

## Abstract

Tart Montmorency cherries (MC) are a particularly rich source of anthocyanins and other polyphenols that have been shown to elicit antioxidant, anti-inflammatory and vasomodulatory actions. The current study aimed to determine the influence of chronic MC supplementation on cognitive function and mood. In a 3-month double-blinded, placebo-controlled parallel study, middle-aged adults (mean ± sd: 48 ± 6 years) were randomly assigned to either 30 ml twice daily of MC (*n* 25) or the same amount of an isoenergetic placebo (*n* 25). Cognitive function and mood were assessed before and after supplementation using a computerised cognitive task battery and visual analogue scales. Cerebral blood flow was also monitored by near-infrared spectroscopy during the task battery, and questionnaires were administered to determine subjective sleep and health status and plasma metabolomics were analysed before and after supplementation. After 3 months, the MC resulted in higher accuracy in digit vigilance (mean difference: 3·3, 95 % CI: 0·2, 6·4 %) with lower number of false alarms (mean difference: −1·2, 95 % CI: −2·0, −0·4) compared with the placebo. There was also a treatment effect for higher alertness (mean difference: 5·9, 95 % CI: 1·3, 10·5 %) and lower mental fatigue ratings (mean difference −9·5, 95 % CI: −16·5, −2·5 %) with MC. Plasma metabolomics revealed an increase in a number of amino acids in response to MC intake, but not placebo. These data suggest an anti-fatiguing effect of MC supplementation as well as the ability to improve sustained attention during times of high cognitive demand, this could be related to changes in amino acid metabolism.

Cognitive decline is the deterioration of cognition that typically occurs with age. Moreover, progressive cognitive decline is implicated in the pathophysiology of neurodegenerative diseases and mood disorders^(1,2)^. Deteriorations in cognitive function happen gradually, commencing in early adulthood and progressing more rapidly during mid-life^(3,4)^. Reduced cognitive function is among the most feared aspects of growing older in the UK, and cognitive failure is the cause for 40 % of admissions to institutional care^(5)^. Hence, maintaining good cognitive function and mental health is important for healthy ageing^(6)^. Given the global ageing population and the inherent economic, personal and societal burdens related to poor cognition, delaying cognitive ageing, reducing the disease risk trajectory and preventing neurodegenerative diseases have become a research priority. Neurodegenerative diseases and mood disorders have some commonality in the underpinning mechanisms that might be related to increased exposure and impaired ability for defence mechanisms to resist oxidative stress and inﬂammation as well as impaired vascular function and cerebral blood flow (CBF)^(7–9)^. Thus, dietary sources of polyphenols that have been shown to improve these factors might serve to maintain better cognitive function and have consequently become a topic of interest^(10)^. For example, in a recent longitudinal study of middle-aged adults from the Framingham Offspring Cohort, highest compared with the lowest dietary anthocyanin intake was associated with lower risk of developing Alzheimer’s disease and related dementia over a 19·7 year follow-up^(11)^.

Anthocyanins (from the Greek *anthos*, a flower and *kyanos*, dark blue) are a subclass of polyphenols responsible for the red and blue pigmentation in fruits and vegetables^(12)^. Tart Montmorency cherries (MC) are a rich source of anthocyanins and other phytochemicals (e.g. (poly)phenols, carotenoids and indolamines^(13)^) that have been demonstrated to cross the blood–brain barrier^(14,15)^. Tart MC phytochemicals have also been reported to exert anti-neuro-inflammatory properties and to suppress neuronal apoptosis and stimulate pro-survival signalling cascades – mechanisms that might protect against cognitive ageing^(16–18)^. Additionally, anthocyanins have also been shown to upregulate brain-derived neurotrophic factor, a potential mechanism and plausible link between dietary anthocyanin intake and improved cognition, particularly memory^(19,20)^. In accordance, Thangthaeng and colleagues^(18)^ reported improvements in working memory, markers of inflammation and autophagy in aged Fischer rats following 6-week supplementation with MC powder compared with a control. Other possible benefits include the potential for MC anthocyanins to enhance blood flow that could result in improved delivery and uptake of oxygen and glucose to the brain to support optimal cerebral functioning^(21–23)^. Moreover, increased endothelial dysfunction, inflammation and poor sleep are closely associated to depression^(24,25)^, and MC have been shown to have favourable influences on these^(26,27)^ suggesting a putative role in cognitive function and mood.

However, evidence from human trials regarding the influence of cherries on mood and cognition is less consistent. For example, acute cherry intake has not been shown to influence cognitive performance, despite modulating blood flow^(21,28)^. Nevertheless, longer-term cherry supplementation has been shown to improve some aspects of cognitive performance^(29,30)^. Moreover, both aforementioned studies^(29,31)^ were predicated by reductions in systolic blood pressure, suggesting that the vasodilatory properties of the cherries might be, at least partly, driving this response. Despite these findings, at present, no attempt has been made to examine the cerebral haemodynamic response to chronic tart cherry supplementation in response to cognitive tasks. Furthermore, as the only longer-term cherry studies have been in older adults, it is not known whether these findings extend to other populations, and certainly there is evidence that midlife might be a critical period to intervene^(32,33)^. It was therefore hypothesised that MC would improve cognitive function and mood and increase cerebral blood flow. In this context, as part of a larger study, we aimed to determine the influence of 3-month supplementation with MC on cognitive function, mood, sleep, health and cerebral blood flow in middle-aged adults.

## Methods

### Participants

**Fig. 1.**
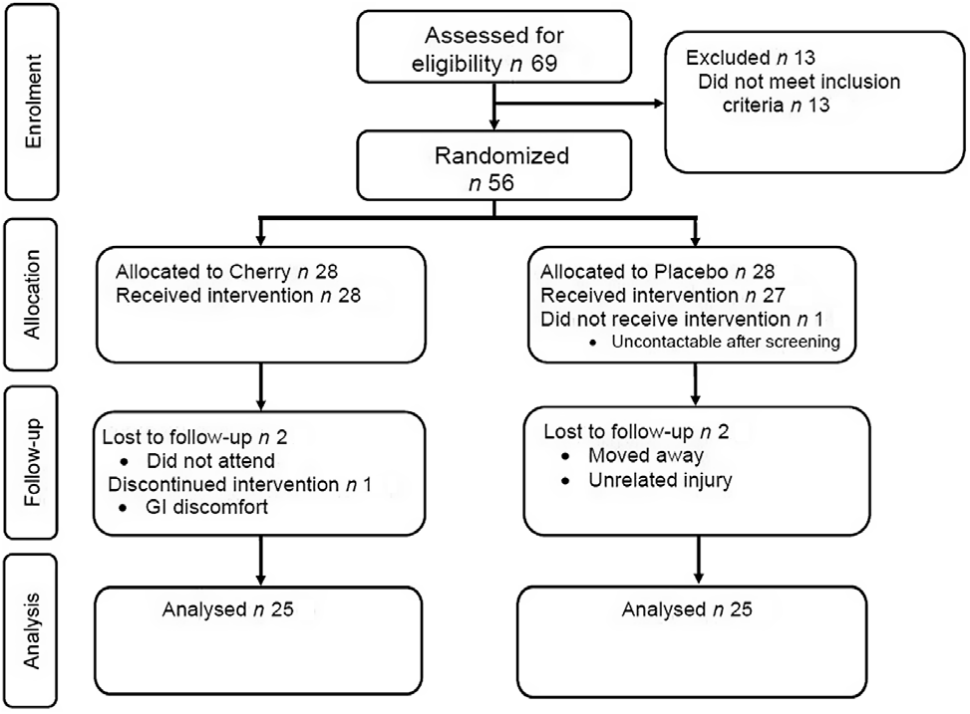
Consort diagram of study enrolment, allocation and analysis.

Fifty non-smoking adults (34/16 males/females; mean ± sd age: 48 ± 6 years and BMI: 27·6 ± 3·7 kg/m^2^) out of fifty-six recruited completed the present randomised, double-blind, placebo-controlled, parallel-arm study (Fig. 1). The participant inclusion and exclusion criteria have been previously reported^(34)^; briefly, to be included in the study, participants had low intake of fruit and vegetable (< 5 servings/d) and low levels of physical activity (≤ 4 h/week of moderate-vigorous activity) and ≥ 1 additional risk factor for type II diabetes^(35,36)^. The study was conducted in accordance with the Declaration of Helsinki and ratified by the University’s Research Ethics Committee prior to participants providing written, informed consent. This study was part of a larger trial examining other health indices associated with polyphenol intake that was registered as a clinical trial (clinicaltrials.gov; NCT04021342); with *a priori* power calculation based on systolic blood pressure as the primary outcome. A *post hoc* power analysis was calculated using G × Power (version 3·1·9·6, Germany) based on the effect size of the significant findings which suggested sufficient power (1-*β* = 1·00; *α* = 0·05; *n* 50) for the current study.

### Procedures

Each participant was required to attend the laboratory on three separate occasions. On the first visit, participants were screened for inclusion/exclusion criteria. If deemed eligible, they were familiarised with the cognitive function tasks using voice recorded instructions^(37)^. Following this, volunteers were randomly assigned using computer-generated plan (randomization.com) 1:1; stratified by sex, to receive either 30 ml, twice daily of MC concentrate (*n* 25) or an isoenergetic placebo (*n* 25) for 3 months. We have previously shown that 60 ml of MC is physiologically relevant and well tolerated^(21,22)^, and other studies had found benefits after bi-daily supplementation strategies^(31,38)^. The sleep, cognitive function, mood, health and cerebral blood flow outcomes were assessed over two experimental visits (visit 2; pre-supplementation) and at 3 months (visit 3; post-supplementation). Visit 2 was preceded by a minimum of a 7-d low anthocyanin run-in, in which berry fruits, red grapes (including extracts/juices) and red wine^(39,40)^ were restricted to ≤ 1 portion per day. Both experimental visits took place at 09.00 ± 1 h and were preceded by an overnight fast (≥ 10 h). Participants were also asked to arrive hydrated and to avoid strenuous exercise, alcohol, nutritional supplements for 24 h and caffeine for 12 h prior. Throughout the study, participants were encouraged to maintain their habitual diet and exercise routines; however, they were asked to refrain from consuming cherries, cherry products or any antioxidant supplements and to limit the aforementioned anthocyanin-rich foods to one or less portion per day throughout the study period. Participants recorded their pre-evening meal before experimental visit one and were asked to replicate this before the second experimental visit. Participants completed an estimated 3-d diet diary (two consecutive weekdays and one weekend day) before, and the International Physical Activity Questionnaire^(41)^ on the day of each experimental visit. Analysis of food diaries and International Physical Activity Questionnaire indicated 100 % adherence to dietary restrictions and no changes in energy intake or physical activity over the study duration (online Supplementary Table 1). Participants total polyphenol (flavonoids, phenolic acids, stilbenes and lignans) and anthocyanin intake was estimated from their 3-d diet diary using Phenol-explorer^(42)^ and is presented in Table 2. Anthocyanin intake was not different between groups, but on average the mean intake of total polyphenols was ∼244 mg/d higher in the cherry group, with the highest polyphenol contribution was from coffee (56 %) and tea (26 %).

**Table 1.** Nutritional composition of treatments per 60 ml

	MC concentrate	Placebo
Energy (kcal)	204	204
Carbohydrate (g)	50	51
Protein (g)	2·2	0·0
Fat (g)	0·0	0·0
TACN (mg)		
Mean	22·2	–
sd *	6·7	
TPC (mg)		
Mean	195·5	21·5
sd †	13·1	2·3

TACN, total anthocyanin content (cyanidin 3 glucoside equivalents); TPC, total polyphenol content (gallic acid equivalents).

* Analysed by pH-differential method (placebo was not analysed because it contained artificial colourant (E129) which causes interference with the assay^(74)^).

† Analysed using a modified Folin–Ciocalteu colorimetric method.

**Table 2.** Baseline characteristics of participants (Mean values and standard deviations)

Variable	Cherry (*n* 25)		Placebo (*n* 25)		*P*-Value
	Mean	sd	Mean	sd	
Age (year)	49	6	47	6	0·465
Sex (m/f)	8/17		8/17		
Stature (cm)	173·8	9·2	173·2	8·9	0·884
Body mass (kg)	82·9	13·9	82·9	12·5	0·968
BMI (kg/m^2^)	27·3	3·8	27·5	3·8	0·570
Anthocyanins (mg/d)	9·7	17·2	17·8	40·5	0·958
Total polyphenols (mg/d)	571·8	244·9	327·9	195·8	0·010
	*n*	%	*n*	%	
Education					
Less than high school	–		–		
High school or equivalent	9	36	12	48	
Bachelor’s degree	9	36	9	36	
Postgraduate degree	7	28	4	16	
Left-handed	3	12	1	4	
Regular use of medication*	6	24	6	24	
Blood pressure	2	8	2	8	
Cholesterol	1	4	2	8	
HRT	1	4	–		
Antidepressant	–		2	8	
Gout	1	4	–		
ADHD	–		1	4	
Asthma	1	4	–		

Attention deficit hyperactivity disorder (ADHD); hormone replacement therapy (HRT).

* Medication stabilised for ≥ 3 months.

### Treatments

The MC concentrate was supplied by Cherry Marketing Institute (Michigan, USA), which was stored at 4°C as directed. Two different batches of the MC concentrate were examined for total anthocyanins and total phenolic content using techniques previously described^(22)^ and found to contain on average 370·2 (sd: 112·2) mg/l of cyanidin-3-glucoside equivalents and 3259·0 (sd: 218·9) mg/l gallic acid equivalents, respectively. The placebo supplement consisted of unsweetened black cherry flavoured Kool-Aid (Kraft Foods Ltd.), dextrose (MyProtein Ltd.), fructose (Sports Supplements Ltd.), lemon juice, artificial food colouring (E129 and E133) and bottled water^(38,43)^. Both drinks were isoenergetic (Table 1) and were packaged in the same polyethylene terephthalate containers, hence similar visual properties. Participants were instructed to dilute each 30 ml serving in ~240 ml of water as recommended. To ensure blinding, participants were given their assigned treatment by a researcher independent to the project along with a 30 ml measuring, blinding was also assessed by treatment guess on the last experimental visit. Treatment compliance was measured by daily tick sheets and return of any unconsumed juice.

### Cerebral blood flow

Changes in cerebral blood flow were assessed using continuous wave near infrared spectroscopy (NIRS) (NIRO-200NX, Hamamatsu Photonics K.K., Japan). Two near-infrared sensors were placed over the left and right frontal lobe region of the forehead corresponding to the International 10–20 system Fp1 and Fp2 electroencephalography (EEG) positions; these signals were averaged to determine cerebral oxygenation. The sensors were secured to the skin using double-sided adhesive tape and shielded from ambient light using an elastic head band. The emitter/optode separation distance of 4 cm. A 5-min rest (which acted as the NIRS baseline for CBF calculations) was taken at each testing session, and data were acquired continuously throughout a cognitive task battery. Output was time stamped at each task segment and averaged over the task period. Baseline adjusted data with respect to the 5 min of NIRS data collected immediately prior to completing the tasks^(44,45)^ were calculated offline. NIRS data are reported as changes in cerebral oxy- (HbO_2_), deoxy- (hHb) and total-(tHb) haemoglobin concentrations.

### Cognitive function, mood, sleep and health assessment

Participants completed the Pittsburgh Sleep Quality Inventory^(46)^ and a short form quality-of-life survey (SF-36;^(47)^) to assess sleep quality and health, respectively. The Pittsburgh Sleep Quality Inventory is a subjective measure of the quality and pattern of sleep over the past 30 d. Questions related to seven domains (subjective sleep quality, sleep latency, sleep efficiency, sleep duration, sleep disturbances, daytime dysfunction and use of medication to assist sleep) and a global score are given, with a higher score indicating ‘poorer sleep’. The SF-36 was used to assess personal perception of general health (average of five components) before and after the intervention. The single item of health changes in the last year was also included to determine any major self-reported changes in health status. Cognitive function and mood measures were assessed using a test battery administered via the Computerised Mental Performance Assessment System (COMPASS, Northumbria University, Newcastle upon Tyne, UK), a purpose-designed software application for the flexible delivery of randomly generated parallel versions of standard and novel cognitive assessment tasks^(22,45)^. The test battery included three tasks: digit vigilance (DV; 3 min), rapid visual information processing (5 min) and *N*-back task (∼3 min). The cognitive tests (described below) were repeated twice in order to induce cognitive fatigue, which was assessed immediately after each battery by a visual analogue scale (VAS). The VAS was presented as ‘mental fatigue’ in which participants had to mark on a line scale anchored ‘not at all’ (left hand end) and ‘very much so’ (right hand end), with higher scores representing more mental fatigue. Participants also completed Bond–Lader VAS^(48)^ before and after the cognitive function tests to assess subjective mood.

### Bond–Lader visual analogue scale

The VAS required participants to indicate how they currently feel ‘at this moment in time’ by clicking, using the mouse, at the appropriate point along a 100 mm scale on screen. Sixteen scales are presented with antonyms at either end, e.g. ‘alert’ *v*. ‘drowsy’, ‘lethargic’ *v*. ‘energetic’ and ‘troubled’ *v*. ‘tranquil’, with these sixteen scores (% along the line towards the right end) combining to create three overall measures of mood factors: ‘alert’, ‘content’ and ‘calm’.

### Digit vigilance

The DV task is a measure of sustained attention and psychomotor speed. A single target digit was randomly selected and constantly displayed on the right-hand side of the screen. A series of single digits appeared on the left-hand side of the screen, one at a time, at the rate of 150 per minute. The participant was required to press the spacebar on the keyboard as quickly as possible every time the digit in the series matched the target digit. Task outcomes included accuracy (%) and reaction time for correct responses (ms) and number of false alarms. This task has been shown to identify age-related declines in attention, and the test-retest correlation coefficient for reaction time is 0·81^(49)^.

### Rapid visual information processing

The rapid visual information processing task is a measure of sustained attention and working memory. The task requires the participant to monitor a continuous series of single digits for targets of three consecutive odd or three consecutive even digits. The digits are presented on the computer screen one at a time at the rate of 100 per minute in pseudo-random order, and the participant responds to the detection of a target string by pressing the spacebar on the keyboard as quickly as possible. Task outcomes included number of target strings correctly detected (%) and average reaction time for correct detections (ms) and number of false alarms. The test-retest correlation for these is (> 0·70) in older adults and has been reported as reliable in the detection and monitoring of cognitive deficits^(50)^.

### N-Back

The three-back task measures working memory and memory capacity. The task requires participants to indicate whether the letter presented on screen was also presented three letters previously in the letter sequence. Participants are required to respond by pressing buttons corresponding to ‘yes’ or ‘no’ on the keyboard, to each letter, as quickly as they can. Participants were presented with forty-five stimuli (letters); however, the task is dependent on speed (i.e. slower reaction times will result in a lengthier task). The task outcomes included accuracy of correct yes responses (%) and reaction time for correct yes responses (ms). The test-retest correlation coefficient for this task has been reported to be 0·73 for accuracy and 0·81 for reaction time, respectively^(51)^.

### Metabolomics protocol

Non-targeted metabolomics was performed on plasma samples. Fasted venous blood samples were collected in lithium-heparin vacutainers (Becton, Dickinson and Company, USA). Due to blood sampling error, samples were only available for thirty-eight participants (*n* 19 for MC and placebo group) for both time points, baseline and 3 months. These were centrifuged at 3000 ***g*** (4°C) for 10 min, and the plasma aliquoted and stored at –80°C.

The plasma samples were defrosted on ice and extracted using a biphasic Folch extraction methodology as follows: 100 ul of plasma samples were extracted in 300 ul of 2:1 chloroform/methanol solution. The samples were vortexed for 1 min and then allowed to incubate on ice for 30 min. Next, 50 ul of optima grade LC/MS water was added to solution-induced phase separation and vortexed for 30 s, and the samples were incubated on ice for additional 10 min. The extraction buffer was then centrifuged at 3000 rpm at 4°C for 15 min, 100 ul of the aqueous layer was collected and filtered via 0·22 micron cellulose filter and transferred to 1·5 autosampler vials with 200 ul microinsert. Quality controls samples were also made by pooling 10 ul of each sample together.

Hydrophilic liquid interaction chromatography metabolite profiling of the plasma samples was performed on a Thermo Scientific (Hemel Hempstead, UK) Vanquish Liquid Chromatography; the chromatographic separation system was connected to IDX high-resolution mass spectrometer. The hydrophilic liquid interaction chromatography positive and negative data sets were processed via Compound Discoverer 3·2 according to the following settings: Untargeted metabolomic workflow: mass tolerance 10 ppm, maximum shift 0·3 min, alignment model adaptive curve, minimum intensity 1^e6^, S/N threshold 3, compound consolidation, mass tolerance 10 ppm and retention time tolerance 0·3 min. Database matching was performed using Thermo scientific m/z cloud databased with a similar index of 70 % or better MS2 spectra. Those metabolites that could be matched (*n* 174) and had a relative standard deviation of 30 % or less within the quality controls were retained for analysis.

The data set was autoscaled and cube root transformed using Metaboanalyst 5.0 software^(52)^ before preforming detailed multivariate and univariate analysis including PCA that was used for identification of outliers. Partial least squares discriminant analysis was used to test for discrimination between sample MC group at baseline and 3 months. The relative metabolite abundance of the metabolites from the MC with variable importance in projection (VIP) factor > 1 was then compared with placebo. The PCA identified two outlier samples (online Supplementary Fig. 1), which were removed before analysis.

### Statistical analysis

All data were analysed using IBM SPSS statistics (v 26.0 for Windows; SPSS), and measures are reported as means ± standard deviation (sd) in tables and standard error (se) in figures unless otherwise stated. Baseline characteristics were compared by Wilcoxon signed-rank test where data were continuous and treatment guess analysed by *χ*^2^ test. Outcome data were cleaned by generating box plots for each outcome variable to identify potential outliers. Values that were more than one and a half and three deviations from the interquartile range were identified as outliers, and extreme outliers, respectively^(53)^, which were removed. Despite familiarisation with the cognitive function tasks, some participants did not perform the tasks correctly (e.g. by pressing the wrong button); therefore, these were removed before data cleaning. The number of participants analysed for each variable can be found in the corresponding tables and figures.

Health (SF-36) and sleep (Pittsburgh Sleep Quality Inventory) data were analysed using the MIXED procedure in SPSS with treatment (cherry juice/placebo) and visit (pre, post) as fixed factors and participant number as a random factor. The post-dose cognitive and mood outcome measures were modelled using the MIXED procedure in SPSS which included the respective baseline values as a covariate and the terms treatment (cherry juice/placebo) and repetition (1, 2) as fixed factors and participant number as a random factor.

The NIRS data were separated into epochs for each task adjusted for resting baseline data (5 min prior). The task length was fixed for the DV (180 s) and rapid visual information processing (300 s), but NIRS data from the N-Back test were truncated so that the same amount of data were analysed for all participants. The epochs were averaged across the two channel hemispheres. If the participant’s data had been omitted from the cognitive function task (for all variable, i.e. accuracy, reaction time and false alarms), the epoch was excluded from analysis for that task. The resting pre-task-adjusted post-dose NIRS outcome measures were modelled using the MIXED procedure in SPSS which included the respective baseline pre-task-adjusted values as a covariate and the terms treatment (cherry juice/placebo) and task epochs (1–6) as fixed factors and participant number as a random factor. Sidak adjusted *post hoc* comparisons were then carried out between cherry juice and placebo as appropriate.

## Results

The baseline demographics of the cohort were similar regarding age, height, weight and BMI, (*P* > 0·05). A full list of demographics including education, left-handed and medication use can be found in Table 2. The study was successfully blinded (*P* = 0·386), and the mean (± sd) self-reported treatment compliance was 94 ± 15 %.

### The effect of Montmorency cherries on cerebral blood flow

After 3-month supplementation, there was no treatment or treatment × epoch interaction effects for HbO_2_, hHb or tHb concentrations assessed by NIRS during any of the tasks (online Supplementary Fig. 2).

### The effect of Montmorency cherries on sleep and health

Overall sleep duration across both visits was higher in the MC group (mean difference: 24·2, 95 % CI: 4·8, 43·6 min: F = 6·15, *P* = 0·015), main effect of treatment. After 3 months, sleep duration had decreased in the MC group 13·8 min and increased in the placebo group 11·6 min, but there was no interaction (F = 1·69, *P* = 0·197). There were no differences between treatments after 3 months for subjective sleep assessed by the Pittsburgh Sleep Quality Inventory or general health and health change assessed by SF-36 (Table 3).

**Table 3.** Subjective sleep quality assessed by Pittsburgh sleep quality inventory (PSQI) and health assessed by short form-36 before and after supplementation with tart Montmorency cherry concentrate or an isoenergetic placebo (Mean values and standard deviations)

	*n*	Cherry		Placebo		Mixed model	
		Mean	sd	Mean	sd	Effect	*P*-value
Sleep Latency (min)	49						
Baseline		16·3	9·4	17·6	9·6	T	0·672
3 months		20·8	14·6	21·7	13·5	T × V	0·932
Sleep Duration (min)*	50						
Baseline		434	63	398	28	T	0·015
3 months		421	54	409	38	T × V	0·197
Habitual Sleep Efficiency (%)	50						
Baseline		90·0	7·0	86·8	7·1	T	0·308
3 months		84·7	8·5	84·5	10·9	T × V	0·380
Global PSQI score	49						
Baseline		4·0	1·6	5·0	1·8	T	0·092
3 months		4·3	1·6	4·5	1·9	T × V	0·278
General health (%)	49						
Baseline		67	19	71	18	T	0·462
3 months		68	13	69	15	T × V	0·569
Health change (%)	47						
Baseline		58	12	58	14	T	0·269
3 months		56	11	51	14	T × V	0·391

Effects are treatment (T) and treatment by visit interaction (T × V).

* Significant difference between treatments (*P* < 0·05).

### The effect of Montmorency cherries on cognitive performance and mood

**Fig. 2.**
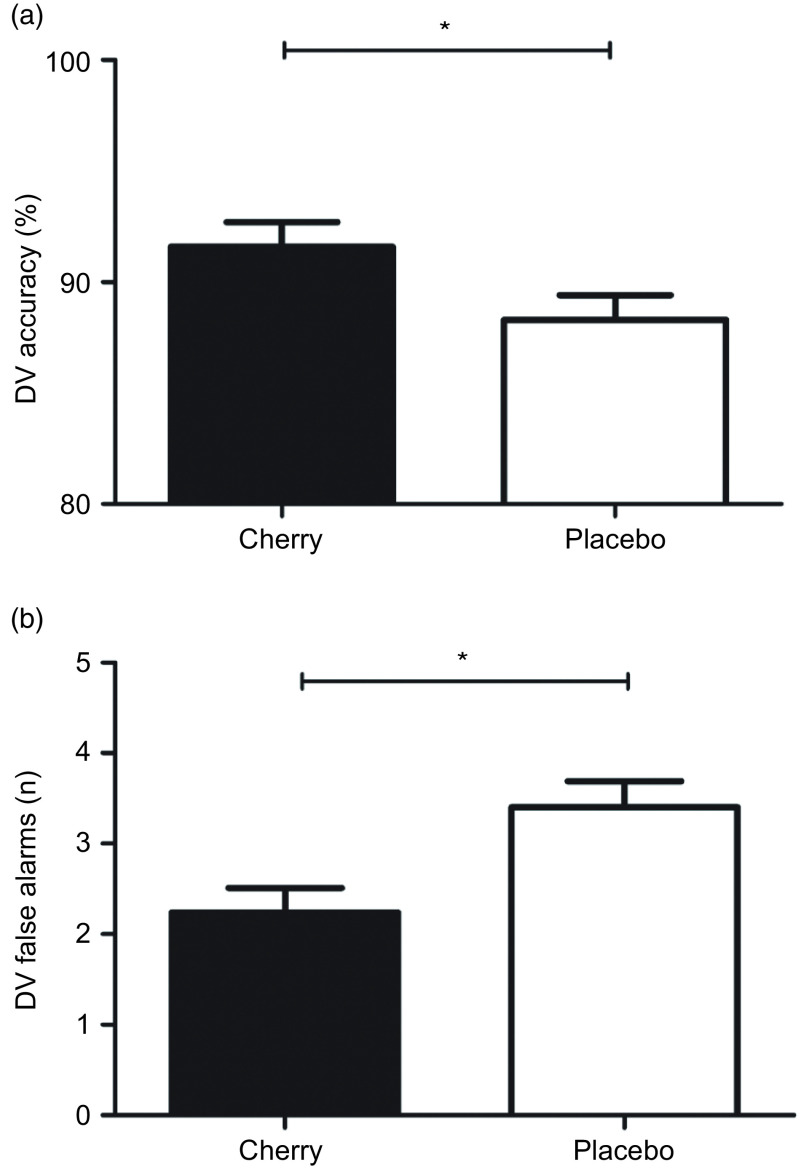
Estimated marginal means and standard error (se) for post-treatment digit vigilance (DV) accuracy (A; *n* 45) and false alarms (B; *n* 41). *P < 0·05 between treatments.

Across repetitions, post-supplementation DV accuracy was higher (mean difference: 3·3, 95 % CI: 0·2, 6·4 %: F = 4·57, *P* = 0·035; Fig. 2(a)), and number of false alarms was lower (mean difference: −1·2, 95 % CI: −2·0, −0·4: F = 8·49, *P* = 0·005; Fig. 2(b)) when adjusted for baseline with MC compared with the placebo. There was no treatment or interaction effects between treatments for any other cognitive function variables (Table 4).

**Table 4.** Cognitive function tasks before and after supplementation with tart Montmorency cherries or an isoenergetic placebo (Mean values and standard deviations)

Measure	Treatment	*n*	Baseline				3 months					
			Rep 1		Rep 2		Rep 1		Rep 2		Mixed model	
			Mean	sd	Mean	sd	Mean	sd	Mean	sd	Effect	*P*-value
DV accuracy (%)*	Cherry	45	94·1	5·9	88·2	13·1	95·7	3·5	88·6	11·6	T	0·035
	Placebo		93·0	6·0	84·7	14·7	89·0	9·8	85·9	10·3	T × R	0·294
DV RT (ms)	Cherry	46	477·8	37·8	492·5	39·5	477·4	33·9	495·3	38·8	T	0·166
	Placebo		473·5	31·4	500·1	37·2	484·7	37·5	511·4	34·2	T × R	0·619
DV FA (*n*)*	Cherry	41	2·5	1·9	2·5	2·3	1·4	1·3	3·1	2·3	T	0·005
	Placebo		2·5	1·9	3·8	1·9	3·6	2·8	4·5	2·8	T × R	0·182
RVIP accuracy (%)	Cherry	42	57·2	17·3	63·0	18·3	63·0	20·9	71·5	15·3	T	0·194
	Placebo		50·4	18·7	53·8	17·2	56·3	21·8	56·7	20·5	T × R	0·765
RVIP RT (ms)	Cherry	41	555·6	62·1	568·1	50·6	555·5	53·5	549·6	35·0	T	0·539
	Placebo		557·9	63·9	540·1	47·3	536·4	51·7	545·1	37·1	T × R	0·351
RVIP FA (*n*)	Cherry	44	5·9	3·8	2·8	1·3	5·5	5·2	3·8	2·6	T	0·955
	Placebo		4·9	3·2	5·2	3·8	4·3	3·1	4·7	2·6	T × R	0·786
3-Back accuracy (%)	Cherry	40	80·2	5·4	85·3	8·4	79·7	9·1	85·4	9·0	T	0·608
	Placebo		76·9	9·3	81·8	8·8	77·5	9·4	83·3	7·9	T × R	0·962
3-Back RT (ms)	Cherry	40	1110	231	980	234	1111	248	942	195	T	0·930
	Placebo		946	239	901	205	1054	286	920	247	T × R	0·653

DV, digit vigilance; FA, false alarm; RVIP, rapid visual image processing; RT, reaction time; Rep, repetition. Effects are treatment (T) and treatment by repetition interaction (T × R).

* Significant difference between treatments (*P* < 0·05).

**Fig. 3.**
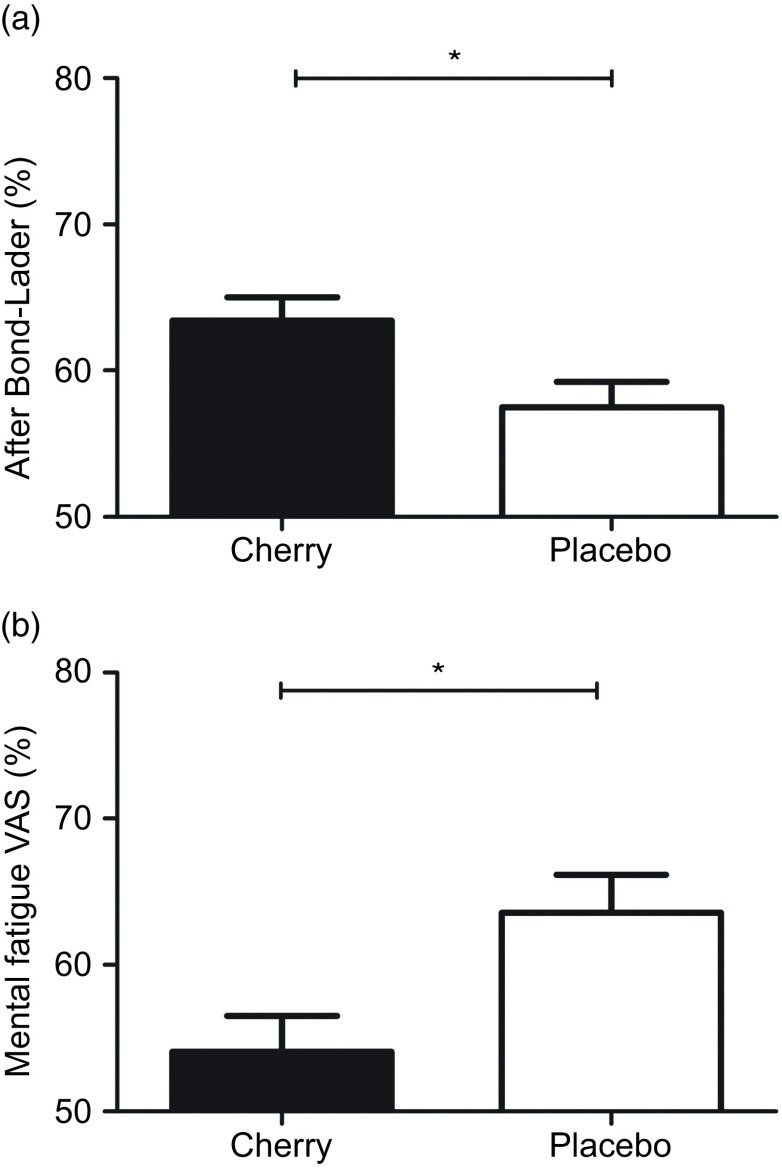
Estimated marginal means and standard error (se) for post-treatment alert Bond–Lader (A; *n* 48) and mental fatigue VAS (B; *n* 46). *P < 0·05 between treatments.

After 3 months, the alert Bond–Lader was higher in the MC (mean difference: 5·9, 95 % CI: 1·3, 10·5 %: F = 6·42, *P* = 0·013; Fig. 3(a)), main effect of treatment. Similarly, post-supplementation mental fatigue VAS was significantly lower (mean difference –9·5, 95 % CI: –16·5, –2·5 %; Fig. 3(b)) in the MC group (F = 7·21, *P* = 0·009). There was no effect of the treatment on calm or content Bond–Lader (Table 5).

**Table 5.** Mood and visual analogue scale measures before and after supplementation with tart Montmorency cherries or an isoenergetic placebo (Mean values and standard deviations)

Measure	Treatment	*n*	Baseline				3 months				Mixed model	
			Rep 1		Rep 2		Rep 1		Rep 2		Effect	*P*-value
			Mean	sd	Mean	sd	Mean	sd	Mean	sd		
Alert (%)*	Cherry	48	71·8	9·5	52·6	14·8	69·3	11·3	59·7	18·5	T	0·013
	Placebo		71·8	11·1	47·8	14·2	65·6	15·3	43·2	14·8	T × R	0·093
Content (%)	Cherry	48	77·5	9·2	68·1	12·6	81·0	7·9	74·4	12·2	T	0·166
	Placebo		76·8	10·9	60·5	18·9	75·8	13·8	66·1	17·9	T × R	0·783
Calm (%)	Cherry	49	74·2	9·6	57·3	13·5	76·0	13·1	64·7	15·3	T	0·699
	Placebo		65·1	18·0	54·4	13·1	74·8	13·7	61·8	18·4	T × R	0·657
Mental fatigue (%)*	Cherry	46	53·5	18·1	72·3	10·5	48·8	21·9	56·0	22·9	T	0·009
	Placebo		59·0	15·1	72·4	21·7	56·6	13·3	64·3	24·6	T × R	0·257

Repetition (rep). Effects are treatment (T) and treatment by repetition interaction (T × R).

* Significant difference between treatments (*P* < 0·05).

### The effect of Montmorency cherries on plasma metabolome

**Fig. 4.**
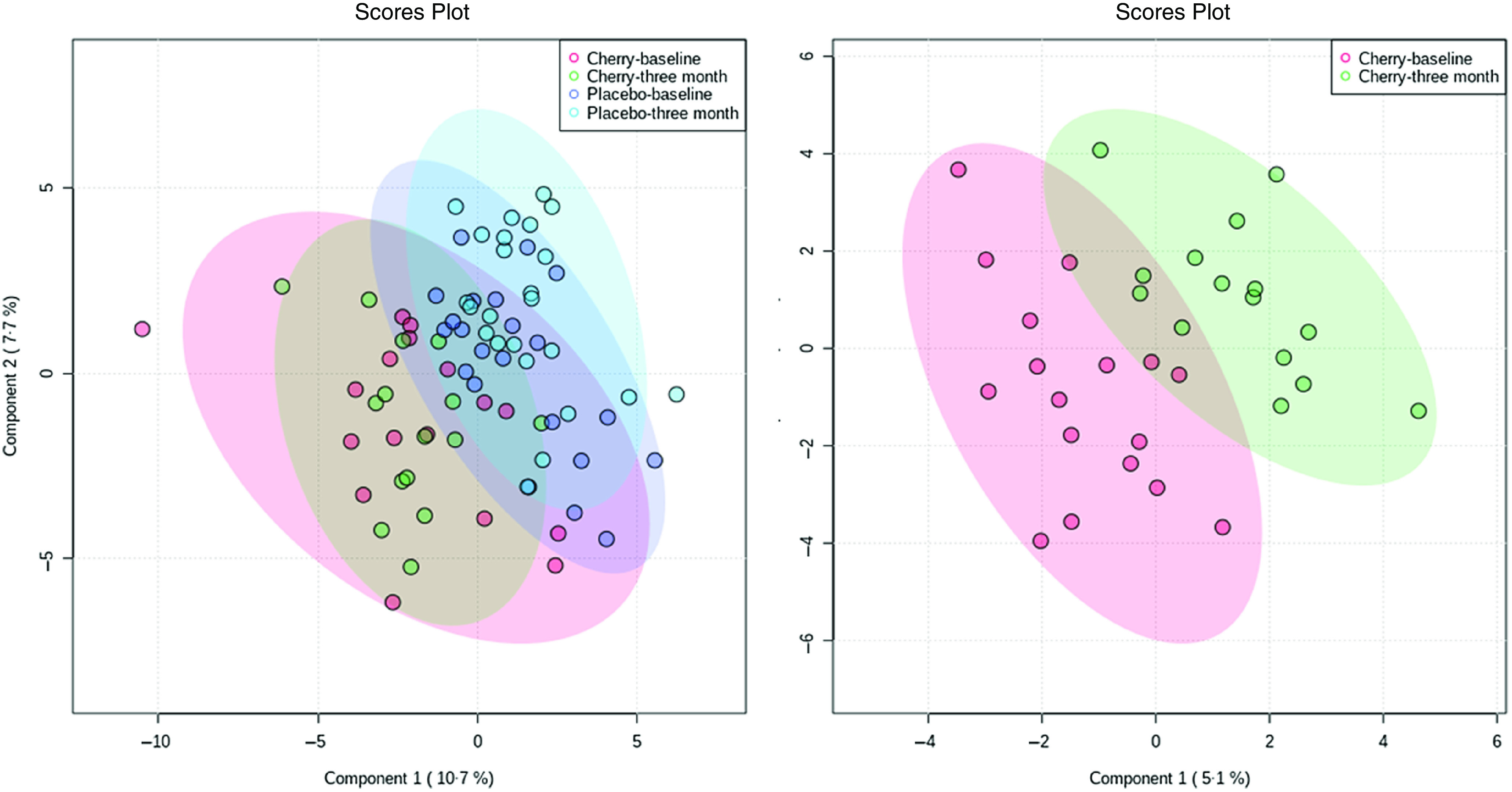
Partial least squares discriminant analysis (PLS-DA) for all treatments (left) and cherry group only (right).

**Fig. 5.**
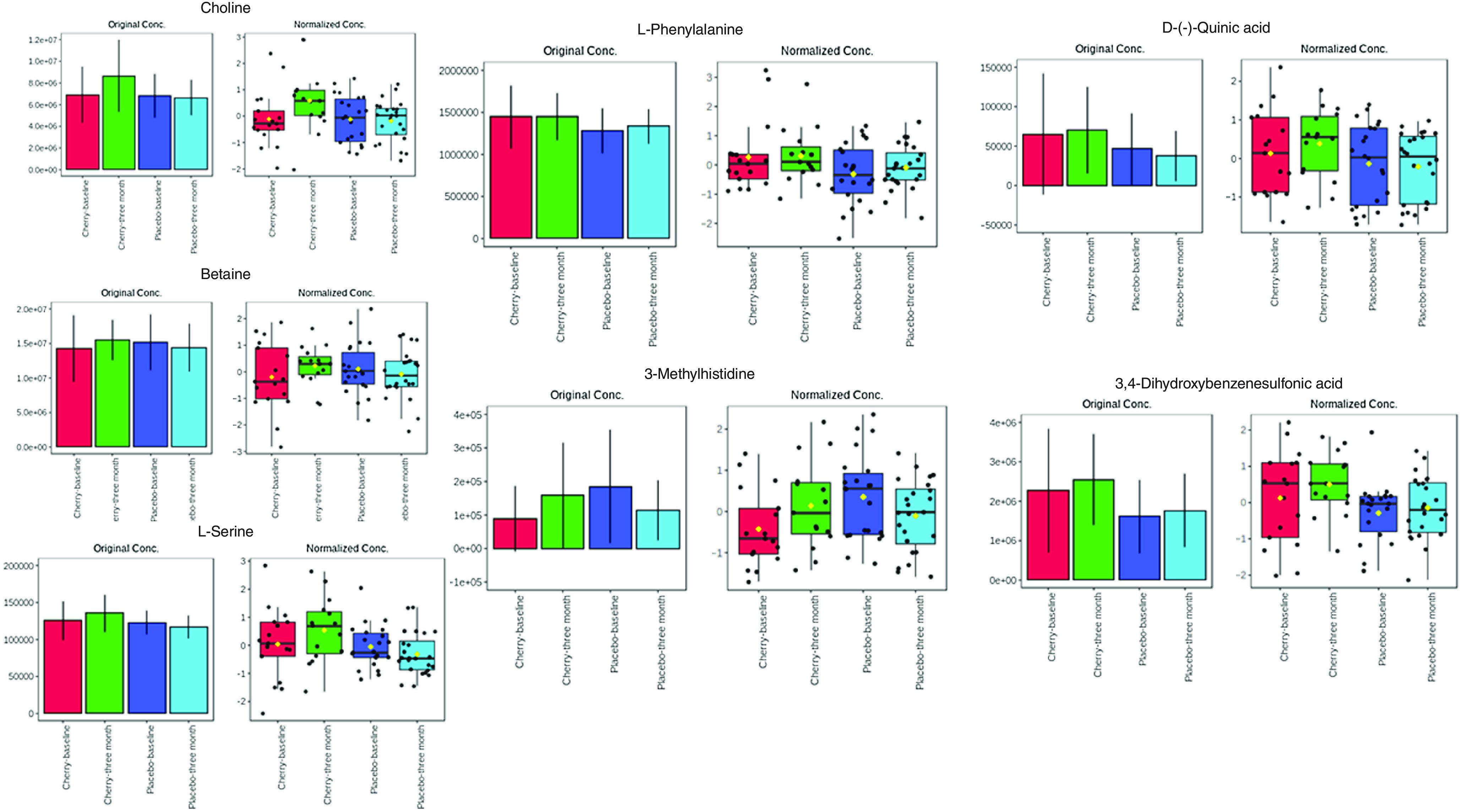
Original and normalised concentration of metabolites upregulated in the cherry but not placebo group post-supplementation.

The partial least squares discriminant analysis for all treatments and MC only at baseline and 3 months are presented in Fig. 4, demonstrating a change in plasma metabolome after supplementation with MC. In total, thirty-five database matched metabolites were shown to be different after 3-month supplementation with MC (VIP > 1; online Supplementary Fig. 3). Polyphenol metabolites, quinic acid and 3,4-dihydroxybenzenesulfonic acid as well as several amino acids; 3-methylhistidine, L-phenylalanine, betaine, L-serine, choline upregulated after post-supplementation with MC but not placebo, Fig. 5.

## Discussion

The main finding of this study was that tart Montmorency cherries have a positive impact on cognitive performance and perceptions of fatigue and alertness and upregulate plasma amino acids, with no influence on CBF, sleep or health. In the current study, MC improved sustained attention measured by DV. Both sweet^(29)^ and tart^(31)^ cherries have been shown to improve aspects of cognitive function following 12-week supplementation in older adults, including sustained attention; however, it is currently unknown whether this is a result of improved CBF or due to the potential neuroprotective properties of tart cherry anthocyanins^(17)^. Therefore, we measured blood flow with NIRS placed over the prefrontal cortex, but no changes in cognitive function or CBF in response to MC intake were observed. There is no directly comparable study, and hence this represents the first study to determine cognitive performance and NIRS in response to chronic supplementation of MC. Although our research group has shown that an acute bolus of MC can influence CBF^(22)^, we did not observe any influence following chronic supplementation in the current study. This is likely to be attributable to the vasomodulatory properties of the cherries coincide with peak plasma concentrations of anthocyanin metabolites, which are rapidly metabolised and/or excreted^(21)^. It is therefore conceivable that changes in vascular function are relatively transient with the bioavailability of the phytonutrients and hence any possible changes from the previous day had passed. This is consistent with our finding that 3 month supplementation had no influence on vascular function variables after an overnight fast^(34)^. Moreover, the data in the present study are in line with previous studies that reported that both resveratrol^(45)^ and *Sideritis scardica*^(54)^ supplementation induced acute, but not chronic changes in CBF parameters measured by NIRS. Furthermore, in a recent review of the influence of polyphenols on CBF, changes following longer-term supplementation were only apparent in studies using MRI, highlighting the potential difficulty in detecting changes in CBF^(55)^. For instance, Bowtell *et al.*^(56)^ reported regional changes in brain perfusion measured by MRI and improved cognitive performance following 12-week supplementation with anthocyanin-rich blueberry concentrate. In the current study, we used continuous wave NIRS and the limitations surrounding this are well documented^(57,58)^, namely it only measures relative changes in cerebral activation and CBF as opposed to the measurement of absolute, quantifiable amounts of haemoglobin present within the cortex. Furthermore, NIRS was measured on the prefrontal cortex and is not representative of changes elsewhere within the cerebral cortex and subsequently could be an area for future research to explore.

In the current study, MC supplementation resulted in lower self-reported mental fatigue and higher alertness. Since these effects were mirrored with increased accuracy and reduced false alarms in the DV task, it would appear that the anti-fatiguing effects of MC could potentially enhance attention and protect against errors. Moreover, this sustained attention could be beneficial in various daily tasks, such as driving and working^(59)^. Only one other study has examined the influence of MC on these aspects of mood, in which an acute bolus of MC had no effect^(22)^, even though CBF was modulated suggesting this might not be the driving mechanism. Other studies have suggested that polyphenol-rich foods such as cocoa might influence mood after chronic, but not acute intake^(60,61)^, albeit the potential underlying mechanisms are yet to be elucidated. As part of an exploratory analysis for mechanistic understanding, we analysed the plasma metabolome of the participants before and after supplementation. Three-month supplementation of MC resulted in the upregulation of some phenolic acid metabolites (e.g. quinic acid^(62)^ and 3,4-dihydroxybenzenesulfonic acid) that were not apparent in the placebo. Interestingly, we also found that MC supplementation upregulated phenylalanine (a precursor to tyrosine) and histidine metabolism in line with a previous small pilot study^(63)^. These amino acids have also been shown to be modulated after short-term intake of red wine and grape polyphenols, which the authors speculate might be due to polyphenols effecting colonic protein fermentation or changing microbial amino acid metabolism^(64)^ that are related to prebiotic actions. There was also upregulation of choline, betaine and serine, which might represent modulation of cholinergic metabolism and is important for attention and cognition^(65–67)^, and histidine supplementation has been shown to improve feelings of mental fatigue^(68)^. The ability for MC to modulate amino acids related to neurotransmitters and cognitive function in the current study has limited comparability to other studies, but importantly is supported by some previous data^(69)^, and warrants further investigation to understand the potential mechanisms of action.

There were no differences found in sleep measures between groups after the intervention, as assessed by the Pittsburgh Sleep Quality Inventory. This contradicts previous research that showed tart cherries, due to their melatonin content, improved sleep quality^(27,43,70)^. However, this is likely because of the use of a questionnaire in this study rather than any objective measures of sleep quality. For example, previous research reported improved sleep efficiency and total sleep time measured by actigraphy, following 7-d consumption of MC, but the same measures collected by questionnaires did not differ^(27)^. Importantly, there were no substantial changes in the sleep patterns of participants, but future studies might employ more quantitative markers. Similarly, other subjective, but nonetheless validated measures like the Bond–Lader and mental fatigue VAS might need to be considered in a similar light.

Other limitations within the current study include that baseline polyphenol intake was different between groups. Second, as discussed elsewhere^(34)^, the sugar content of MC and potential variability between batches need to be carefully considered in future research designs^(71)^. Third, based on emerging evidence, it is speculated that (poly)phenols might be beneficial compounds; however, it should be acknowledged that MC contains other phytochemicals that could synergistically have an effect^(30,72,73)^. Lastly, metabolomics was only conducted on compounds that could be matched to the database, but it should be acknowledged that other important compounds could have been missed. Notwithstanding, due to the low number of adverse events, good compliance levels reported and no effect on subjective health as assessed by the SF-36, it is reasonable to suggest that 60 ml/d of MC is a safe and tolerable intervention. Moreover, to date this is the first study to determine the effect of chronic supplementation of MC on cognitive function in middle-aged adults. To the best of our knowledge, this is also the only study in tart Montmorency cherries to concurrently examine the CBF mechanism and plasma metabolome to cognitive outcomes following longer-term supplementation. Therefore, this study provides insight into the effects of tart cherries on cognitive performance, fatigue and mood in middle-aged adults, which might be related to the ability to modulate amino acid metabolism and provides a platform for future research.

In conclusion, the current study reports higher sustained attention, self-reported alertness and lower mental fatigue following supplementation with MC. The intervention also upregulated amino acids that might indicate a potential underlying mechanism. These data provide new information that bioactive foods that are rich in anthocyanins and other (poly)phenolic compounds can have an anti-fatiguing effect during periods of high cognitive demand, which are beneficial in daily tasks requiring vigilance.

## Supplementary material

For supplementary material accompanying this paper visit https://doi.org/10.1017/S0007114522000460. click here to view supplementary material
