# Supplementary material for: Polyphenol-rich tart cherries (Prunus Cerasus, cv Montmorency) improve sustained attention, feelings of alertness and mental fatigue and influence the plasma metabolome in middle-aged adults: a randomised, placebo-controlled trial
Source: Br J Nutr. 2022 Feb 3;128(12):2409–20. doi: 10.1017/S0007114522000460 (PMC9723490; doi:10.1017/S0007114522000460)
Supplement: Supplementary file 1 [file S0007114522000460sup001.docx]

**Supplementary material**

**Table 1.** Dietary intake and exercise at baseline and study end

|  | **Cherry**  N=25 | **Placebo**  N=25 | **ANOVA** | | | |
| --- | --- | --- | --- | --- | --- | --- |
|  |  |  |  | Treatment | Time | Interaction |
| **METs (min/week)** |  |  |  |  |  |  |
| Baseline | 2462 ± 2038 | 1953 ± 1527 |  | 0.267 | 0.959 | 0.961 |
| 3 months | 2464 ± 2336 | 1978 ± 1217 |  |  |  |  |
| **Sitting time (h)** |  |  |  |  |  |  |
| Baseline | 6.6 ± 3.1 | 6.7 ± 3.0 |  | 0.856 | 0.852 | 0.486 |
| 3 months | 6.7 ± 2.8 | 6.3 ± 3.0 |  |  |  |  |
| **Energy (Kcal)** |  |  |  |  |  |  |
| Baseline | 1921 ± 340 | 1896 ± 430 |  | 0.997 | 0.212 | 0.673 |
| 3 months | 1977 ± 439 | 1987 ± 420 |  |  |  |  |
| **Carbohydrates (%)** |  |  |  |  |  |  |
| Baseline | 43.8 ± 6.1 | 41.7 ± 4.9 |  | 0.587 | <0.001 | 0.297 |
| 3 months | 47.6 ± 9.0^a^ | 47.3 ± 8.3^a^ |  |  |  |  |
| **Fat (%)** |  |  |  |  |  |  |
| Baseline | 35.8 ± 5.9 | 36.7 ± 4.9 |  | 0.689 | 0.019 | 0.648 |
| 3 months | 33.3 ± 6.5 | 33.0 ± 7.3^a^ |  |  |  |  |
| **Protein (%)** |  |  |  |  |  |  |
| Baseline | 16.7 ± 3.2 | 19.9 ± 3.5* |  | 0.176 | 0.007 | 0.001 |
| 3 months | 16.8 ± 4.4 | 16.1 ± 3.5^a^ |  |  |  |  |
| Mean ± SD  ^a^Significantly different from baseline (P <0.05): * significantly different between groups (P <0.05) | | | | | | |

**
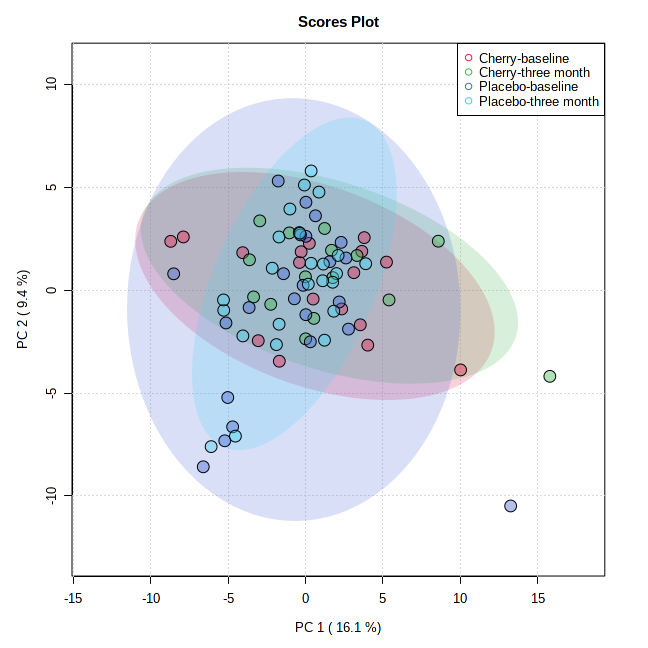
**

**Figure 1.** Principal component analysis (PCA) of all groups before and after supplementation

**
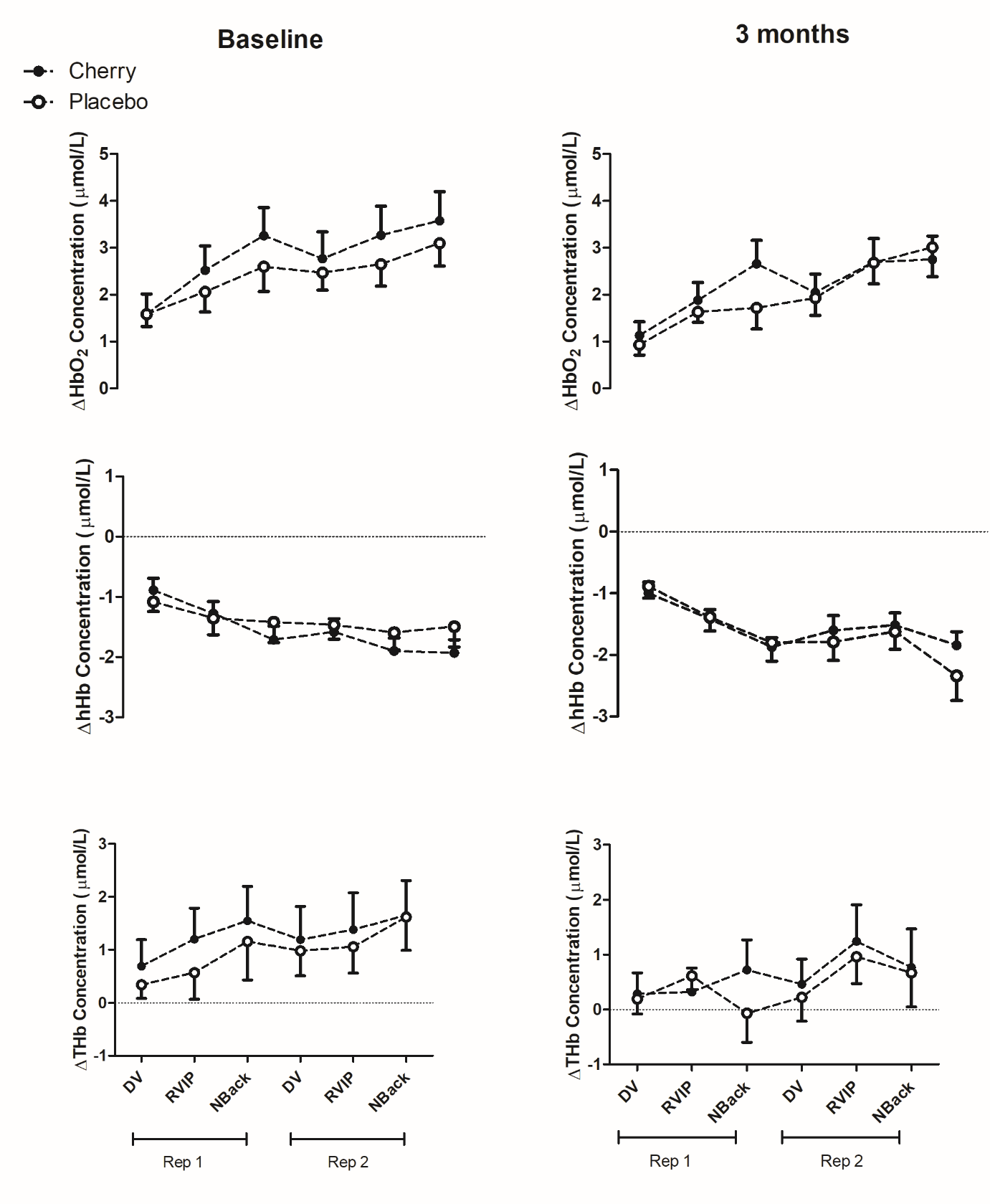
**

**Figure 2.** The pre-task adjusted oxygenated (HbO_2_; top panel; n = 44), deoxygenated (hHb; middle panel; n = 42) and total- haemoglobin (THb; bottom panel; n = 41) concentrations for each task repetition pre-supplementation (left) and post-supplementation (right). Rep = repetition

**
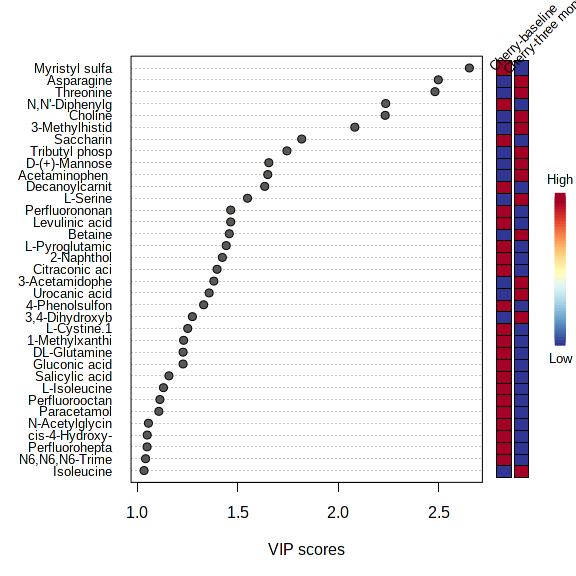
Figure 3.** Heat map of metabolites that were discriminatory between baseline and 3-month supplementation with Montmorency tart cherries (Variable Importance in Projection (VIP) factor >1).
